# Value of high-sensitivity C-reactive protein in low risk chest pain observation unit patients

**DOI:** 10.1186/1865-1380-4-37

**Published:** 2011-06-24

**Authors:** Deborah B Diercks, J Douglas Kirk, Seif Naser, Samuel Turnipseed, Ezra A Amsterdam

**Affiliations:** 1 Department of Emergency Medicine, University of California, Davis Medical Center, Sacramento, CA, USA; 2 Division of Cardiology, University of California, Davis Medical Center, Sacramento, CA, USA

## Abstract

**Objective:**

High-sensitivity C-reactive protein (hs-CRP) rises with cardiac injury/ischemia. We evaluated its efficacy in aiding in the identification of an acute coronary syndrome (ACS) in patients (pts) admitted to the chest pain unit (CPU) for possible ACS.

**Methods:**

Retrospective study of all patients admitted to the CPU with chest pain who underwent hs-CRP testing as part of their CPU evaluation from January 2004 to October 2008. Patients were low risk for ACS (compatible symptoms, nondiagnostic initial ECG, and negative cTnI). ACS was diagnosed by positive functional study, cardiac catheterization, or cardiac event during 30-day follow-up. Positive hs-CRP was defined based on local laboratory levels (>1.0 mg/l or >3.0 mg/l), and population-based and prior study values >2.0 mg/l. Chi-square analysis was performed, and odds ratios (OR) are presented. Multivariate analysis was done to determine whether hs-CRP was independently associated with the diagnosis of ACS. Cardiac risk factors, demographics, and diagnosis of ACS were included in the model. Medians with IQR are presented for continuous data. Ninety-five percent confidence intervals are presented where applicable.

**Results:**

A total of 958 patients had hs-CRP testing as part of their CPEU evaluation. Excluded from the analysis were 39 patients lost to follow-up. The final cohort comprised 478 (52%) women and 441 (48%) men with a median age of 56 (IQR 48-64). ACS was diagnosed in 128 (13.4%). The median cohort hs-CRP value was 2.2 mg/l (IQR 0.7, 5.8) and 2.3 mg/l (IQR 0.6, 5.9) in those with and without ACS, respectively. In the multivariate analysis hs-CRP was not independently associated with the diagnosis of ACS (0.99; 95% CI 0.98 - 1.01).

**Conclusion:**

In large patient cohort managed in a single-center CPU, measurement of hs-CRP did not enhance the diagnostic accuracy for ACS. Routine hs-CRP as a diagnostic tool should not be recommended in the CPU setting.

## Introduction

The management of patients presenting to the emergency department (ED) with chest pain is a continuing challenge. Of primary concern is recognition or exclusion of an acute coronary syndrome (ACS), which is initiated with risk stratification that encompasses a protocol-driven approach that begins with the patient's history, physical examination, and electrocardiogram, (ECG) [1]. This method provides early evidence of the presence or absence of myocardial ischemia/injury. In those patients with negative findings, which indicate low or moderate clinical risk depending on individual clinical characteristics, normal cardiac injury markers further reduce the probability of ACS and subsequent adverse clinical events. The injury markers currently utilized are the cardiac troponins, the myocardial band (MB) of creatinine kinase, and myoglobin. The findings of normal confirmatory evaluation such as a treadmill stress test in addition to this negative initial evaluation identifies a group of patients that can be safely discharged from the ED with early follow-up [1].

Numerous biomarkers have been investigated for the rule in the pathophysiology of ACS, including markers of inflammation [2,3]. Acute inflammation is an important factor in ACS through its role in coronary plaque disruption and other aspects of the pathophysiology of this syndrome. The association of inflammation with ACS is reflected by an increase in multiple inflammatory markers that can be detected as acute phase reactants. High-sensitivity C-reactive protein (hs-CRP) is a non-specific marker of inflammation that has been shown to have prognostic significance in patients with coronary artery disease [4,5]. This property has led to the evaluation of hs-CRP as a diagnostic tool in patients presenting to the ED with chest pain, and the diagnostic utility of hs-CRP in this setting has been variable depending on the patient populations studied [6-9]. In this investigation we evaluated the diagnostic accuracy of hs-CRP in low risk patients evaluated for ACS in our chest pain unit (CPU) in order to determine whether this marker can aid in the discrimination of those with and without ACS.

## Methods

This is a secondary analysis of prospective data from a single urban tertiary medical center CPU from 1 April 2004 to 31 October 2008. The study was approved by our institutional review board.

### Patient population

#### Inclusion criteria

All patients with a chief complaint of chest pain or an anginal equivalent referred to our CPU were eligible for this study. Our CPU accepts patients who have been evaluated by the ED physician for suspicion of ACS, but are considered low risk based on a normal cardiac injury marker profile and a normal, non-diagnostic, or abnormal but unchanged ECG [10]. Depending on the individual patient risk, patients are further risk stratified by a variety of methods that include obtaining serial ECGs and cardiac injury markers, and performance of confirmatory testing, such as stress electrocardiography, myocardial perfusion imaging, stress angiography, and coronary angiography. Serum hs-CRP was ordered in these patients at the discretion of the CPU physician. Only those patients who underwent hs-CRP testing were included in this analysis.

### High-sensitivity C-reactive protein

We utilized hs-CRP by Beckman SYNCHRON LX. The manufacturer's threshold for an abnormal value intermediate for cardiovascular events was 1.0 mg/l to 3.0 mg/l and high risk >3.0 mg/l.

### Outcome measure

The outcome measure of ACS was defined by ischemia on ECG cardiac marker elevation, or an abnormal confirmatory test indicating the presence of coronary artery disease (CAD). Lack of ACS was determined by negative diagnostic testing or absence of a cardiac event (death, myocardial infarction, or revascularization) during the 30-day follow-up period. Patients lost to follow-up and/or who did not undergo a diagnostic test were excluded from the analysis.

### Statistical analysis

#### Hs-CRP threshold

We evaluated the thresholds for intermediate risk and high risk based on manufacturer recommendations. The dichotomous threshold for hs-CRP was created using the ROC curve. We also evaluated the hs-CRP threshold ≥2.0 mg/l based on a recent large clinical trial [11]. Continuous data were assessed for normality, and median interquartile ranges are presented. Measures of association were performed (odds ratio) and diagnostic test characteristics determined. Sensitivity, specificity, and likelihood ratios were calculated and 95% confidence intervals (CI) presented for local laboratory hs-CRP thresholds as defined above, population threshold defined by the ROC curve, and the value used in clinical trials.

Logistic regression was performed to determine if hs-CRP was independently associated with the diagnosis of ACS. Covariates associated with the diagnosis of ACS were entered into the regression analysis (age, gender, cocaine or methamphetamine use, past medical history of diabetes, hypertension, hypercholesterolemia, family history of myocardial infarction, tobacco use, prior history of CAD, and a normal electrocardiogram). Hs-CRP was evaluated as a continuous variable (Table 1). All analyses were performed on Stata 9.0, (College Station, TX).

**Table 1:** Regression analysis for the association of hs-CRP with the diagnosis of acute coronary syndrome

Variable	Adjusted OR (95% CI)
Age (year)	**1.03 (1.01-1.05)**
Male gender	**1.71 (1.12-2.61)**
Prior CAD	**2.80 (1.77-4.41)**
Family history of CAD	1.33 (0.86-2.08)
Hypertension	1.34 (0.78-2.33)
Cholesterol	**1.68 (1.03-2.78)**
Diabetes mellitus	0.97 (0.62-1.51)
Tobacco	1.43 (0.92-2.24)
Cocaine	0.59 (0.16-2.15)
Amphetamine	2.2 (0.79-6.32)
Normal ECG	0.71 (0.47-1.07)
hs-CRP mg/l	0.99 (0.98-1.01)

CAD, coronary artery disease; ECG, electrocardiogram; hs-CRP, high sensitivity C reactive protein

## Results

A total of 958/3,173(30.2%) patients had hs-CRP testing as part of their CPU evaluation. Of these 958 patients, 40 (4.1%) were lost to follow-up and therefore excluded from the study. The final cohort comprised 918 patients, including 478 (52%) women and 441 (48%) men with a median age of 56 years (IQR 48-64 years). Demographic data are presented in Table 2. Confirmatory testing methods used to diagnose ACS included: stress electrocardiography (n = 327), myocardial perfusion scintigraphy (*n *= 251 stress, *n *= 59 rest), stress echocardiography (*n *= 83), and coronary angiography (*n *= 178). The final outcome of ACS was diagnosed in 128 (13.4%).

**Table 2:** Patient demographic data and factors associated withdiagnosis of ACS

Variable	**ACS**,	**No ACS**,
	*N *= 128 (%)	*N *= 790 (%)
Age (years) (mean; SD)	(56.2; 12.0)	(61.4; 12.2)
Male gender	78 (60.9)	362 (45.8)
Prior disease	60 (46.8)	119 (15.0)
Family history of CAD	42 (32.8)	232 (29.3)
Hypertension	106 (82.8)	525 (66.4)
Cholesterol	98 (76.5)	412 (52.1)
Diabetes mellitus	43 (33.6)	228 (28.8)
Tobacco	53 (41.4)	268 (33.9)
Cocaine	4 (3.1)	30 (3.8)
Amphetamine	7 (5.4)	21 (2.6)
Normal ECG	52 (40.6)	456 (57.7)

ACS, acute coronary syndrome; SD, standard deviation; CAD, coronary artery disease; ECG, electrocardiogram

**Figure 1:**
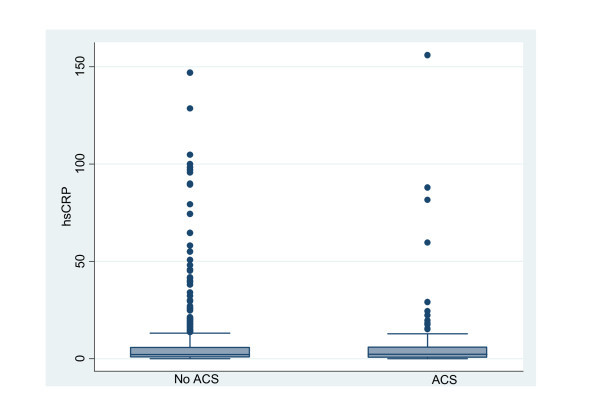
**hs-CRP values in presence of acute coronary syndrome**.

**Figure 2:**
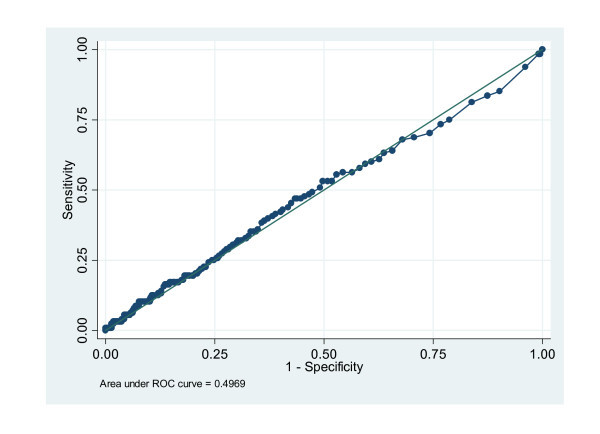
**ROC curve for the diagnostic accuracy of hs-CRP**.

The median cohort hs-CRP value was 2.2 mg/l (IQR 0.7, 5.8) and 2.3 mg/l (IQR 0.6, 5.9) in those with and without ACS, respectively (Figure 1). Based on the population-based ROC curve, no threshold for hs-CRP to predict the diagnosis of ACS could be defined (Figure 2). Using a threshold defined in prior clinical trials, an elevated hs-CRP had a sensitivity, specificity, positive likelihood ratio, and negative likelihood ratio for ACS of 53.1%, 50.3%, 1.05, and 0.99, respectively. In addition, measures of diagnostic accuracy are presented for all studied thresholds in Table 3.

**Table 3:** Measures of diagnostic accuracy for the diagnosis of acute coronary syndrome

Hs-CRP thresholds	Sensitivity	Specificity	Likelihood ratio positive	Likelihood ratio negative
≥1.0 mg/l	68% (59.1%-75.9%)	32% (28.9%-35.3%)	1.00 (0.88-1.14)	0.99 (0.76-1.31)
>3.0 mg/l	43.8% (35.2%-52.8%)	58.5% (55.0%-61.9%)	1.05 (0.85-1.3)	0.96 (0.81-1.13)
≥2.0 mg/l	53.1% (49.6%-67.7%)	50.3% (46.8%-53.9%)	1.05 (0.76-1.45)	0.99 (0.94-1.04)

The 40 patients who were excluded from the study because they were lost to follow-up differed slightly from the overall cohort. These patients had a lower prevalence of both hypertension and elevated cholesterol than the study cohort.

## Discussion

In this study of low-moderate risk patients admitted to our CPU who underwent hs-CRP testing, we found little diagnostic utility for detection of ACS as defined by a positive confirmatory study or subsequent adverse cardiac event during a limited post-discharge interval. This study adds to the limited information on hs-CRP as a diagnostic tool in patients presenting to the ED with chest pain.

The initial evaluation of CRP in the setting of acute evaluation, did not utilize a high-sensitivity assay. Prior studies evaluating the use of CRP in the acute setting vary with the risk of disease, length of follow-up, and type of CRP testing utilized. Magadle et al. measured CRP levels in 226 patients referred to the ED with chest pain who were followed for 1 year and in whom the outcome measure was occurrence of coronary events. Based on a diagnostic threshold for CRP of 25 mg/l, sensitivity for the diagnosis of ACS was 93%, specificity 65%, and negative predictive value 96% [8]. This study excluded 100 patients because of medical conditions other than CAD that may elevate CRP. Mitchell et al. evaluated a convenience sample of 414 patients admitted to a CPU in whom the prevalence of ACS was 1.7%. Utilizing a multi-marker panel, they found that the negative likelihood ratio for CRP was 0.79 (95% CI 0.4-1.01) [6]. Liyan et al. evaluated 113 patients presenting to the ED within 12 h of chest pain onset. Ninety patients had a final diagnosis of ACS [11]. Using a population-based threshold for CRP of ≥3.16, the sensitivity for diagnosis of ACS was 70% and specificity 74% [12]. Despite the differences in the study populations, the relatively consistent negative results of the foregoing studies suggest the diagnostic accuracy of CRP adds little to the assessment of these patients.

Our study evaluated the use of a contemporary hs-CRP at a single point in time. Lozona et al. evaluated a subgroup of 191 ED patients with an inconclusive cause of chest pain and determined the diagnostic value of a change in two measurements of hs-CRP. In the cohort of 191 patients, 38 had a diagnosis of ACS. All patients completed a CPU protocol with functional testing or coronary angiography. There was no difference in the initial hs-CRP values between the groups of patients with and without CAD [7]. However, in those patients with an increase in hs-CRP from presentation to 24 h later, the sensitivity, specificity, likelihood ratio (LR)+, and LR- for the diagnosis of ACS were 95 (95% CI 81, 98), 40% (95% CI, 32, 47), 1.57 (95% CI, 1.33, 1.83), and 0.13 (95% CI 0.04, 0.44), respectively [7]. This study suggests that changes in hs-CRP may be more reflective of an acute coronary process, although the specificity was unsatisfactory.

## Limitations

This study is a retrospective analysis of existing clinical data. Our CPU protocol does not mandate that all patients undergo confirmatory diagnostic testing. Therefore, not all patients had diagnostic tests for CAD performed, and this may have underestimated the true incidence of ACS in our patient population. Patients admitted to our CPU have risk factors for coronary artery disease, such as diabetes, and therefore this population may have increased CRP values when compared to a patient population that lacks these risk factors for cardiac disease. Although hs-CRP is on our standard order form for the CPU, not all patients underwent hs-CRP testing, which could have resulted in selection bias. Physicians were not blinded to the results of the hs-CRP value, and it is possible that medications with potential anti-inflammatory actions, such as statins, may have been initiated as a result of a high value, which could have altered the risk of subsequent cardiac events within the 30-day follow-up period. In addition, we evaluated hs-CRP only in the context of its diagnostic value for ACS. It is possible that the true value of hs-CRP may be related to mortality or identification of an alternative diagnosis as prior studies have shown that hs-CRP has prognostic value [11].

## Conclusion

In our large patient cohort, managed at a single-center CPU, measurement of hs-CRP did not enhance the diagnostic accuracy for detecting ACS. Based on our study, we do not recommend routine measurement of hs-CRP as a diagnostic tool in this patient population.

## Competing interests

Deborah B. Diercks: Consultant: Sanofi Aventis, Daiichi Sankyo, Abbott Vascular Institutional Research Support: Beckman Coultier, Nanosphere, Board of Directors: Society of Chest Pain Centers and Providers

Bryn Mumma: No competing interests

## Authors' contributions

DD drafted the initial chapter. Both DD and BM edited and contributed to the final content of the review article.
